# Whole-Genome Duplication and Host Genotype Affect Rhizosphere Microbial Communities

**DOI:** 10.1128/msystems.00973-21

**Published:** 2022-01-11

**Authors:** Julian C. B. Ponsford, Charley J. Hubbard, Joshua G. Harrison, Lois Maignien, C. Alex Buerkle, Cynthia Weinig

**Affiliations:** a Department of Botany, University of Wyoming, Laramie, Wyoming, USA; b Program in Ecology, University of Wyoming, Laramie, Wyoming, USA; c Marine Biological Laboratory, Josephine Bay Paul Center, Woods Hole, Massachusetts, USA; d Laboratory of Microbiology of Extreme Environments, UMR 6197, Institut Européen de la Mer, Université de Bretagne Occidentale, Plouzane, France; e Department of Molecular Biology, University of Wyoming, Laramie, Wyoming, USA; University of Dundee

**Keywords:** *Arabidopsis thaliana*, whole-genome duplication, multinomial modeling, plant-microbe interactions

## Abstract

The composition of microbial communities found in association with plants is influenced by host phenotype and genotype. However, the ways in which specific genetic architectures of host plants shape microbiomes are unknown. Genome duplication events are common in the evolutionary history of plants and influence many important plant traits, and thus, they may affect associated microbial communities. Using experimentally induced whole-genome duplication (WGD), we tested the effect of WGD on rhizosphere bacterial communities in Arabidopsis thaliana. We performed 16S rRNA amplicon sequencing to characterize differences between microbiomes associated with specific host genetic backgrounds (Columbia versus Landsberg) and ploidy levels (diploid versus tetraploid). We modeled relative abundances of bacterial taxa using a hierarchical Bayesian approach. We found that host genetic background and ploidy level affected rhizosphere community composition. We then tested to what extent microbiomes derived from a specific genetic background or ploidy level affected plant performance by inoculating sterile seedlings with microbial communities harvested from a prior generation. We found a negative effect of the tetraploid Columbia microbiome on growth of all four plant genetic backgrounds. These findings suggest an interplay between host genetic background and ploidy level and bacterial community assembly with potential ramifications for host fitness. Given the prevalence of ploidy-level variation in both wild and managed plant populations, the effects on microbiomes of this aspect of host genetic architecture could be a widespread driver of differences in plant microbiomes.

**IMPORTANCE** Plants influence the composition of their associated microbial communities, yet the underlying host-associated genetic determinants are typically unknown. Genome duplication events are common in the evolutionary history of plants and affect many plant traits. Using Arabidopsis thaliana, we characterized how whole-genome duplication affected the composition of rhizosphere bacterial communities and how bacterial communities associated with two host plant genetic backgrounds and ploidy levels affected subsequent plant growth. We observed an interaction between ploidy level and genetic background that affected both bacterial community composition and function. This research reveals how genome duplication, a widespread genetic feature of both wild and crop plant species, influences bacterial assemblages and affects plant growth.

## INTRODUCTION

Plant-microbe interactions can exhibit a positive feedback cycle in which changes in the microbiome affect plant phenotype and, conversely, the phenotype of the plant host alters microbial community composition (1–3). Rhizosphere bacteria, in particular, affect many aspects of plant performance, such as increasing access to nutrients (4), relieving abiotic and biotic stress (5), and promoting growth (6). Even slight changes in the rhizosphere microbiome can affect host plant phenotype (7). For instance, Korir et al. found that increased abundance of one taxon, Bacillus megaterium, enhanced nitrogen access and growth of Phaseolus vulgaris under field conditions (8). Wholesale changes in the abundance of taxa composing rhizosphere microbiomes also affect plant performance (9, 10). For example, in Arabidopsis thaliana, differences in microbial community composition, attributable to past or nearby plant communities, strongly influenced host growth (11, 12).

A plant host’s genetic background can also affect the composition of microbiomes comprising thousands of taxa (13–15). For instance, over a range of environmental conditions, host genotype in maize explains on average ∼19% of the variance in relative abundance of root microbial taxa (16). Among host genotypes, allelic variation segregating at loci with diverse functions could potentially contribute to differences in rhizosphere community composition. The contribution of specific host plant loci and pathways has been demonstrated experimentally using genetic knockouts or transgenic overexpression (17, 18). Genetic manipulation to shift the plant circadian clock by ±4 h explained ∼22% of the variance in rhizosphere bacterial communities among experimental *Arabidopsis* lines (12). However, the number of studies characterizing causal genetic factors is small, and the extent to which specific host genetic and genomic features alter the assembly and function of microbial communities remains largely unknown, despite the demonstrably important effect of the rhizosphere microbiome on host plant performance.

Whole-genome duplication (WGD) is one genetic feature of host plants that could potentially influence microbial community composition. WGD can drastically affect host phenotype, including inducing changes in life history and physiology, and can even induce changes at the cellular level (19, 20). WGD occurs naturally in wild populations and is ubiquitous in the evolutionary history of plants and in the domestication history of many crop species (21). For all these reasons, we hypothesize that WGD could have an important influence on the composition of plant microbiomes.

We tested this hypothesis through experimental induction of WGD in *A. thaliana* using colchicine. This compound is used to induce autopolyploidization and create stable lines of tetraploids in *A. thaliana.* The colchicine-induced mutation is reported to produce no genetic changes besides those associated with genome duplication (22). Therefore, the comparison of microbiomes between *A. thaliana* tetraploid genotypes developed from inbred diploid lines allows characterization of the influence of host ploidy level on microbial community composition, without the confounding effects of allelic variation or fixed differences in heterozygosity between tetraploids and diploid progenitors. Shifts in ploidy have a variety of phenotypic consequences in *A. thaliana*, including changes in core metabolic pathways such as the tricarboxylic acid cycle (TCA), malate and citrate concentrations, and potassium uptake (23, 24). For the specific genotypes of *A. thaliana* that we used here (Columbia [Col] and Landsberg erecta [Ler]), cell and organ size, circadian rhythms, biomass, chlorophyll and starch content, and other traits are all known to be influenced by shifts in ploidy (25–27).

In the current study, we tested how WGD affects rhizosphere bacterial composition. Rhizosphere microbes are of particular interest because of the influence they have on their hosts (see above) and because plants are known to shape rhizosphere microbial communities via root exudation of low-molecular-weight organic compounds (28–30), which are known to be influenced by WGD (23). Thus, the potential effects of WGD on many aspects of host phenotype support the hypothesis that rhizosphere microbiomes are influenced by genome duplication. We tested this hypothesis and also examined how WGD-induced changes in bacterial community composition affected plant performance.

## RESULTS

### Sequencing results.

**FIG 1:**
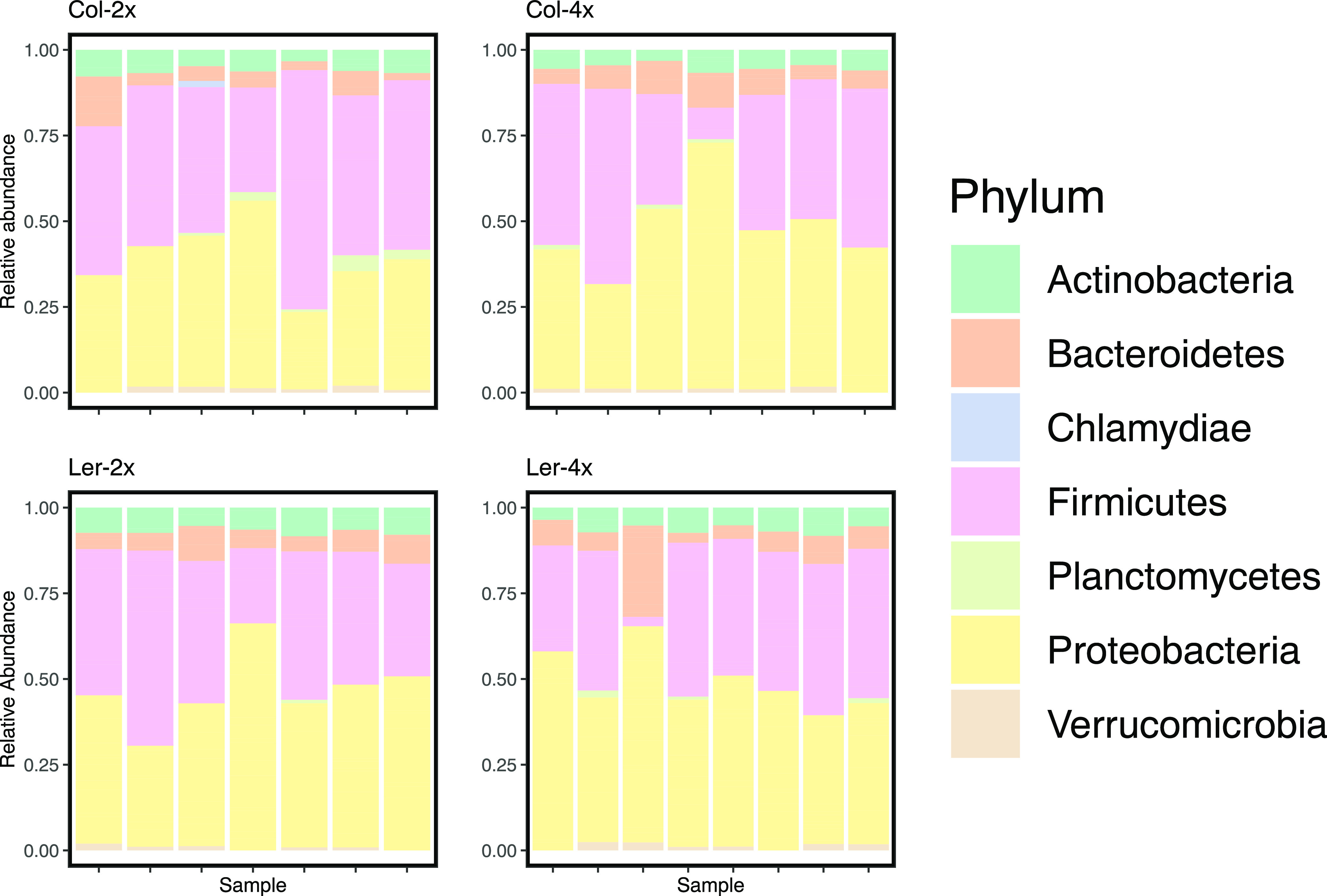
Relative abundance of each rhizosphere associated bacterial phylum in plants with differing genetic backgrounds. Each colored bar represents an individual rhizosphere sample. Phyla are arranged alphabetically. ASVs with a relative abundance of less than 0.05 were removed for graphical clarity.

After quality filtering and removal of nontarget taxa, we recovered 1,647,741 reads, remaining from an initial 3,282,410 raw reads. We retained 36,033 to 143,254 reads per plant rhizosphere (Table S1). In total, there were 2,689 microbial taxa (amplicon sequence variants [ASVs]) present (Fig. 1).

10.1128/mSystems.00973-21.3 TABLE S1 Number of reads at each step of sequence data processing. Download Table S1, DOCX file, 0.01 MB. Copyright © 2022 Ponsford et al. 2022 Ponsford et al. https://creativecommons.org/licenses/by/4.0/ This content is distributed under the terms of the Creative Commons Attribution 4.0 International license.

Genetic background (Col versus Ler) affected both the presence (*P* = 0.021) and abundance of taxa (*P* = 0.016) and explained 5.8% and 7.2% of the variance between groups (in terms of Jaccard and Bray-Curtis dissimilarity matrices) (Table 1), respectively (corroborating results are reported in references 14 and 28).

**TABLE 1:** Effects of plant (versus soil only), plant genotype, and ploidy level on microbial community composition as estimated using PERMANOVA^*a*^

Comparison	Jaccard			Bray-Curtis		
	*P*	*F*	*R* ^2^	*P*	*F*	*R* ^2^
Landsberg vs. Columbia	0.021*	1.665	0.058	0.016*	2.102	0.072
Diploid vs. tetraploid	0.530	0.950	0.0340	0.449	0.958	0.034
Col-2x vs. Col-4x	0.165	1.225	0.0927	0.158	1.342	0.101
Ler-2x vs. Ler-4x	0.978	0.693	0.051	0.959	0.611	0.045
Col-4x vs. all others	0.018*	1.743	0.061	0.013*	2.218	0.0759

a Asterisks indicate *P* value < 0.05.

**FIG 2:**
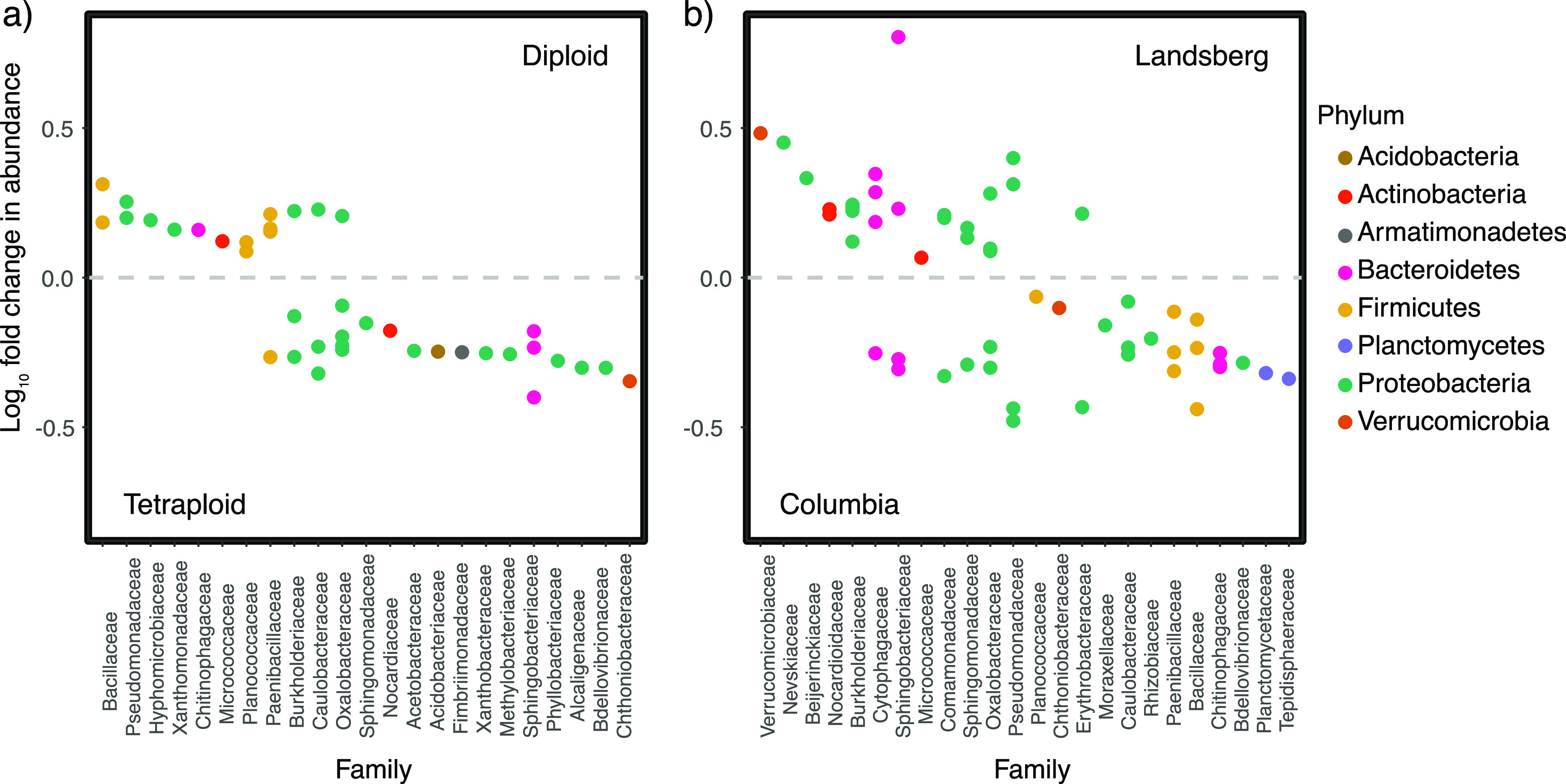
Genotype and ploidy influence rhizosphere bacterial relative abundances. (a) Bacterial families identified as more abundant in the diploid rhizosphere are shown above the gray line, and those more abundant in the tetraploid rhizosphere appear below the gray line. (b) Bacterial families identified as more abundant in the Landsberg rhizosphere are shown above the gray line, and those more abundant in the rhizosphere of Columbia plants appear below the gray line. Log_10_ fold change were calculated from relative abundance estimates obtained through hierarchical Bayesian modeling of read counts (see the text). Points represent individual ASVs within families.

Host genetic background and ploidy level influenced the relative abundances of various bacterial taxa, as demonstrated via the Dirichlet-multinomial model (DMM), though wholesale shifts in the microbiome at the phylum level among treatment groups were not observed (Fig. 1). We identified 25 taxa that were significantly enriched in both Ler genotypes and 29 taxa that were more abundant in the Col genotypes (Fig. 2a). A taxon of the genus *Pedobacter* was the most highly enriched in Ler associated bacterial communities, with an 8-fold increase. In Col bacterial communities, the most enriched taxon was Pseudomonas, with a 4.8-fold increase in abundance. DMM also identified 17 taxa that were significantly enriched in all diploid genotypes. Of these taxa, the most enriched taxon was a member of the genus *Bacillus*, enriched 3.1-fold relative to tetraploid communities. Tetraploid communities’ most enriched member was *Mucilaginibacter*, which was present at a 4-fold-greater relative abundance than in rhizosphere microbiomes in diploids. Twenty-three taxa were significantly enriched across both tetraploid genotypes (Fig. 2b). Our model identified 16 taxa as having higher abundance in the Columbia tetraploid rhizosphere and 30 taxa enriched across the bacterial microbiomes of the other three host genotypes. In Col-4x, a bacterium hypothesized to be *Sphingomonas* had the highest change in abundance at 6-fold. Conversely, a taxon of the genus *Pedobacter* was enriched in all other microbiomes. This taxon appeared at a 5.8-fold-greater frequency in these microbiomes than Col-4x. Across all comparisons, ASVs within the phylum *Proteobacteria* were more commonly differentially abundant than taxa within any other phylum.

When examining shifts in the entirety of the rhizosphere community we observed only subtle effects of ploidy level (permutational multivariate analysis of variance [PERMANOVA]) (Table 1), whether we considered all taxa (Fig. S2) (PERMANOVA, *P* = 0.52 and *P* = 0.456) or only the 742 most abundant taxa, which were represented by >100 reads across all replicates. However, because we observed differential growth effects of Col-4x versus all other rhizospheres, we performed a *post hoc* comparison of the rhizosphere microbiome of the Col-4x genotype to that of other genotypes, including Col-2x. We found that Col-4x differed significantly from all other rhizospheres based on both Jaccard and Bray-Curtis dissimilarities (Table 1) (PERMANOVA, *P* = 0.018 and *P* = 0.013).

10.1128/mSystems.00973-21.2 FIG S2 (a) Principal coordinate analysis of Jaccard dissimilarities (*n* = 29). Rhizosphere community composition does not differ significantly by host plant ploidy level (PERMANOVA, *P* = 0.52) but does differ between genetic backgrounds (Col versus Ler background) (PERMANOVA, *P* = 0.021). (b) Principal-coordinate analysis of Bray-Curtis dissimilarities (*n* = 29). Rhizosphere community abundance does not significantly differ between host ploidy levels (PERMANOVA, *P* = 0.456) but does differ between genetic backgrounds (Col versus Ler background) (PERMANOVA, *P* = 0.020). Both dissimilarity analyses show significant divergence from unplanted soil (PERMANOVA, *P* < 0.01). Download FIG S2, EPS file, 0.2 MB. Copyright © 2022 Ponsford et al. 2022 Ponsford et al. https://creativecommons.org/licenses/by/4.0/ This content is distributed under the terms of the Creative Commons Attribution 4.0 International license.

Shannon’s diversity index revealed no significant differences between genotypes (*P* = 0.911), between ploidy levels (*P* = 0.565), or between ploidy levels nested within genotype (Landsberg, *P* = 0.768; Columbia, *P* = 0.564), based on Welch’s two-sample *t* test. The average diversity across all samples was 4.59 (±0.043; *n* = 29).

### Effects of rhizosphere microbes on plant performance.

**FIG 3:**
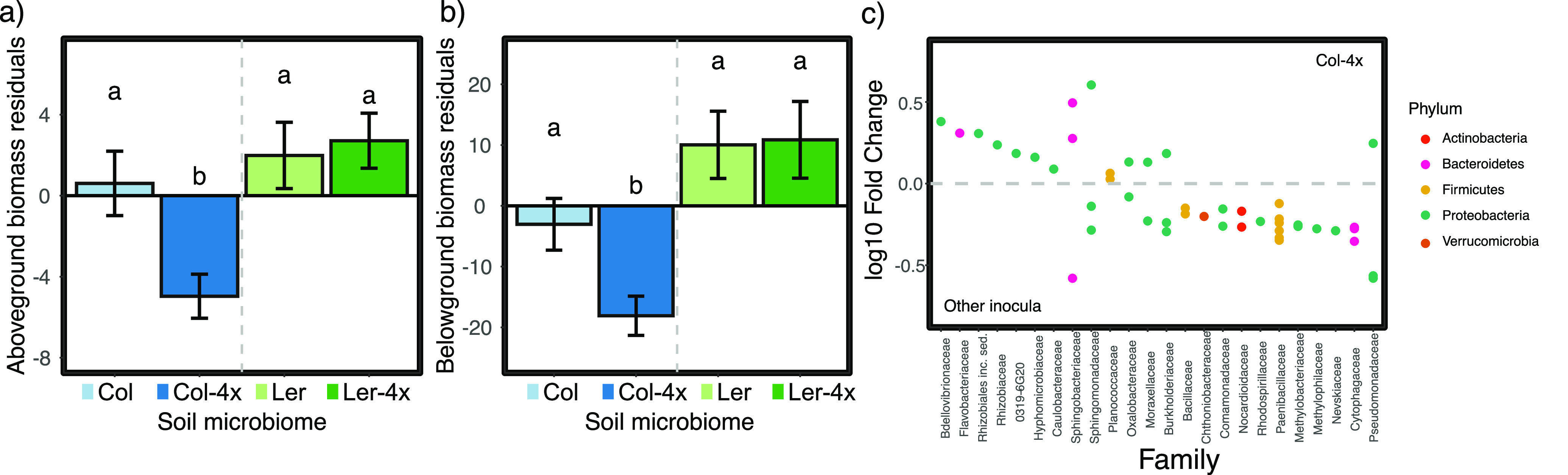
(a and b) Residuals of observed growth differences in above- and belowground biomass for plants grown in soils inoculated with microbiomes shaped by each genotype, with effects of experimental block removed (blocking factors were host genetic background and ploidy level). Bar height represents the mean of residuals; bar color corresponds to soil inoculum across all plant genotypes. Error bars represent standard errors of the means. Lowercase letters denote significant differences among groups as determined by Tukey’s honestly significant difference (HSD) test. All genotypes inoculated with Columbia tetraploid microbiome had significantly reduced above- and belowground biomass. (c) Bacterial families identified as more abundant in the Columbia tetraploid rhizosphere are shown above the gray line. Those more abundant in the rhizosphere of all other treatment groups (inocula) appear below the gray line. The data depicted in this figure are sequencing data describing the rhizospheres from plants in each treatment group and are the data referenced in Fig. 1 and 2. Log_10_ fold changes were calculated from relative abundance estimates obtained through hierarchical Bayesian modeling of rhizosphere associated ASV read counts following processing described in Materials and Methods. Points represent individual ASVs within families.

When microbiomes from plant-conditioned soil were harvested following experiment 1 and used as inoculants in experiment 2, plants grown in soils inoculated with the Col-4x microbiome had significantly lower aboveground and belowground biomass than plants grown in soils inoculated with the Col-2x microbiome (ANOVA, *P* < 0.001 and *P* = 0.012) or microbiomes influenced by either of the two Ler genotypes (Fig. 3a and b) (*P* < 0.001 and *P* < 0.001). The soil microbiome had no effect on phenological characteristics or fruit number (Table S2).

10.1128/mSystems.00973-21.4 TABLE S2 Effect of inoculum shaped by plant genotype on plant performance. Download Table S2, DOCX file, 0.01 MB. Copyright © 2022 Ponsford et al. 2022 Ponsford et al. https://creativecommons.org/licenses/by/4.0/ This content is distributed under the terms of the Creative Commons Attribution 4.0 International license.

## DISCUSSION

Whole-genome duplication (WGD) is estimated to have occurred across 30 to 70% of the angiosperm phylogeny over its evolutionary history and is a common genetic feature of many plant taxa (31). Here, we report that WGD can influence rhizosphere microbiomes and that the effects of host genotype on rhizosphere microbiomes can feed back to influence plant growth.

Specifically, we found that many individual taxa shifted in relative abundance in response to host ploidy level (Fig. 2a). This provides support for the hypothesis that these taxa are directly responsive to genome duplication. Although the causal physiological mechanisms remain unclear, one candidate mechanism is root exudation. Previous studies suggest that root exudates can affect the occurrence and abundance of microbial taxa in the rhizosphere (2, 32) and that these exudates can be influenced by genome duplication (23). Consequently, shifts in root exudates, or other metabolites produced by the plant, in response to WGD could be partially responsible for the association between ploidy and the relative abundance of microbial taxa that we observed (33, 34). This hypothesis awaits further testing.

Given that the number of microbial taxa with a credible response to WGD was in the tens rather than the hundreds or thousands, we conclude that while host ploidy level affects the abundance of some taxa, it may not lead to broad-scale changes in the rhizosphere microbiome. A caveat to this result is that we used soil with a history of *Arabidopsis* occurrence as an inoculant (this is in contrast to prior studies of the *A. thaliana* microbiome [35]). It is possible that WGD could have a stronger influence on microbiota of plants growing in soil with a different history and, thus, with potentially fewer microbes adapted to living alongside *A. thaliana*.

We found that differences in the rhizosphere bacterial microbiome led to differences in host performance (9, 36, 37). Specifically, plants grown in soils inoculated with microbial rhizosphere communities harvested from Col-4x plants had reduced vigor compared to plants grown in soils inoculated with microbial communities from all other genotypes (Fig. 3). While our experiment was not designed to parse the individual effects of specific microbial taxa on host phenotype, we did find an intriguing enrichment of the bacterial species Noviherbaspirillum autotrophicum in the Columbia tetraploid genotype that could plausibly have influenced host growth. *N. autotrophicum* is a facultative autotroph capable of denitrification (38). It is plausible that the greater abundance of *N. autotrophicum* that we observed in tetraploid Columbia plants may have resulted in a decrease in available nitrogen in the rhizosphere, causing the lower overall biomass that we observed for this treatment group.

Regardless of causal mechanism, the reduction of biomass we observed for tetraploid Columbia plants compared to the other genetic backgrounds we considered may have effects on nutrient cycling or plant fitness. This result highlights the likely widespread feedback between plant ploidy level and genetic background, microbiota, and plant phenotype. The implications of this feedback could be particularly notable in mixed populations of diploid and tetraploid individuals (which is common for many plant taxa, including members of *Arabidopsis* [39]), where stressful conditions could be compounded or mitigated by host-associated microbes that are themselves influenced by host genetic background (40). To speculate, it may even be the case that the ASVs enriched (or reduced) in the rhizosphere of tetraploid hosts could partially explain the advantages tetraploids appear to have in stressful environments (41, 42).

### Conclusions.

Given the prevalence of WGD among plants in both natural and agricultural systems, our results highlight a novel mechanism by which plant evolutionary history influences the root-associated microbiome. Moreover, the finding that ploidy level-induced shifts in microbiota were associated with plant phenotypic response suggests ways in which the feedback between host genetic background, microbiome, and phenotype could influence ecosystem-level processes.

## MATERIALS AND METHODS

### Plant material and growth conditions.

To test the effects of whole-genome duplication and plant genetic background on rhizosphere bacterial community composition and plant host performance, we selected two *A*. *thaliana* diploid genotypes: Columbia (Col-2x) and Landsberg erecta (Ler-2x) and their tetraploid counterparts (Col-4x CS922178 and Ler-4x CS3900). Tetraploid genotypes were multiple generations removed from initial colchicine treatment and therefore can be assumed to be mutationally stable (43). In our first experiment, each genotype was planted into sterilized potting mix (Redi-Earth potting mix; Sungro Horticulture, Agawam, MA, USA) and inoculated with a microbial community from the Catsburg region of Durham, NC, USA (36.0622°N, −78.8496°W), a site with a history of *A*. *thaliana* growth. The microbiomes associated with each host plant genotype were characterized by 16S rRNA gene amplicon sequencing in the first experiment and, in a second experiment, tested for effects on growth of a second generation of plants (Fig. S1).

10.1128/mSystems.00973-21.1 FIG S1 Flow diagram of experiments 1 and 2. Download FIG S1, EPS file, 0.3 MB. Copyright © 2022 Ponsford et al. 2022 Ponsford et al. https://creativecommons.org/licenses/by/4.0/ This content is distributed under the terms of the Creative Commons Attribution 4.0 International license.

In both experiments, seeds were surface sterilized using a solution of 15% bleach, 0.1% Tween, and autoclave-sterilized reverse osmosis-purified water (RO H_2_O). This treatment is unlikely to remove microbes that colonize the interior of the seed, but seed-associated microbial assemblages are generally very species poor (44). Seeds were thoroughly rinsed in RO H_2_O to remove any remaining detergents. Seeds were placed in 1.5-ml tubes containing 1 ml RO H_2_O, stored at 4°C for 7 days, and then placed on greenhouse benches in natural 14-h photoperiods to induce synchronous germination (45). On the day root radicles were observed, seeds were transferred to 2-in.-diameter net pots filled with a mixture of autoclaved potting mix and 2 ml of liquid inoculant (described below). To ensure sterility, potting mix was autoclaved on a wet cycle for 60 min at 121°C, allowed to rest for at least 1 h, and autoclaved for another 60 min. No microbes could be cultured on tryptic soy agar medium using serial dilutions of autoclaved soil as the inoculum. It is unclear if autoclaving sufficiently denatures DNA such that it would not occur in our 16S analysis; however, any DNA that remained and was sequenceable would be randomly distributed among samples and not confound treatment. To create inoculants for the first experiment, 60 g of the Catsburg soil was mixed with 540 ml of autoclaved RO H_2_O and sieved through 1,000-μm, 212-μm, and 45-μm sterile sieves to remove nematodes that could potentially affect plant performance (46). The Catsburg site not only has a well-documented occurrence of naturalized *A. thaliana* populations but also has a soil pH approximately identical to that of the potting substrate (5.4 and 5.3, respectively), which minimized potential selection by the common soil matrix on microbial community composition (47–49).

For the second experiment, we used four randomly selected plant-conditioned soil samples from each host genotype to create inocula. Soil for this inocula was harvested concurrent with and in the same manner as rhizosphere samples collected during experiment 1. Soil was combined by genotype, manually homogenized, and used to create the inoculum, as described above. In all experiments, after 10 days of growth, seedlings were thinned to one plant per pot. All experiments were performed at the Williams Conservatory or the Agricultural Experiment Station at the University of Wyoming.

### Experimental design. (i) Experiment 1: effects of host plant genotype and ploidy on microbial community.

To measure the influence of host ploidy on rhizosphere bacterial community composition, 20 replicates of Col-2x, Col-4x, Ler-2x, and Ler-4x were planted in a fully randomized four-block design. To avoid confounding the effects of ploidy with developmental stage, eight rhizosphere samples from each genotype were collected on day 24, by which time all plants displayed visible elongation of the primary inflorescence from the apical meristem (*n* = 29; 1 replicate each of Col-2x, Col-4x, and Ler-2x were lost during processing). Unharvested plants were allowed to grow until senescence to quantify additional traits. To account for variation in microbial communities due to greenhouse conditions and intrinsic variation in soil independent of plant host effects, we collected soil from empty pots containing only soil that were potted simultaneously with our experiment (*n* = 7).

### (ii) Experiment 2: microbial effects on plant performance.

To investigate how host performance is affected by microbiomes associated with specific host genotypes, we used a fully factorial design with four host plant genotypes grown in four host-influenced microbiomes (harvested from the host genotypes in experiment 1). For logistical reasons, we pooled soils from four randomly selected plants from each genotype to create our soil inocula, which we then used to determine the influence of rhizosphere microbes on plant performance. We note that pooling soils from replicate plants from each experimental treatment obscures variation among replicates, and we cannot exclude the possibility that the results we observed were driven by unusual replicate plants within a treatment group. Forty replicates of each plant genotype were grown in sterilized potting mix inoculated with one of the four rhizosphere microbiomes from plant-conditioned soil in a fully randomized block design. Plants were checked daily for bolting and flowering. Upon the first observation of plant bolting (day 24), a subset of plants was collected for above- and belowground biomass (*n* = 382) to measure plant growth and resource allocation. A subset of plants were allowed to senesce, and seeds were harvested to quantify seed mass (*n* = 152). All phenotypic measurements were performed as described by Rubin et al. (50).

### DNA extraction and sequencing.

Rhizosphere samples were collected from the root surface as described in reference 51 by first uprooting plants, manually agitating them, removing loosely adhering soil particles up to 2 mm from the roots, placing roots with closely adhering soil (<2 mm distant from roots) in a buffer of sterile phosphate-buffered saline (PBS) with 0.1% Silwet (Momentive, Waterford, NY, USA) in a 15-ml tube, and vortexing for 10 min on maximum speed. Rhizosphere samples were centrifuged at a relative centrifugal force (RCF) of 3,714, and a total soil mass of no more than 250 mg was transferred from each tube into a sterile Qiagen PowerSoil (Qiagen, Valencia, CA, USA) bead tube; a similar soil mass was collected for the plantless soil controls. DNA was extracted following the manufacturer’s instructions. Extracted DNA was shipped to the Marine Biological Laboratories (Woods Hole, MA, USA) for amplification and sequencing of the V4-V5 (primers 518f and 926r) region of the 16S rRNA gene (52) on an Illumina MiSeq system (paired-end 2 × 250; Illumina, San Diego, CA, USA).

### Sequence and data analysis.

We used Trimmomatic version 0.36 (53) to remove adapters and primer sequences. We used default settings in the R package dada2 (ver. 1.5.8) to filter and trim reads based on quality (forward reads trimmed to 220 bp and reverse reads trimmed to 210 bp), estimate the error rate using 1,000,000 reads, dereplicate reads, infer amplicon sequence variants (ASVs), merge paired-end reads, remove chimeras, and assign unique sequences to taxa using the SILVA 16S database (ver. 128) (54, 55). We removed ASVs corresponding to chloroplasts, mitochondria, and eukaryotes and ASVs for which the SILVA 16S database could not determine a kingdom-level classification. All other ASVs assigned an “NA” taxonomic classification at any level were retained. Next, we ascertained the relative abundance of taxa by quantifying unique sequences and amplicon sequence variants using the default settings in the microbiome R package (56). We used adonis (permutational multivariate analysis of variance using distance matrices) on Jaccard (presence-absence) and Bray-Curtis (abundance) dissimilarities between community samples to characterize the effects of genotype (Col-2x and Col-4x versus Ler-2x and Ler-4x) and ploidy (Col-2x and Ler-2x versus Col-4x and Ler-4x), and calculated Shannon’s diversity index to characterize intra- and intergenotype variation, using the vegan and phyloseq R packages (57–59).

Dissimilarity analyses identify coarse-grained changes in community composition across treatments, which arise from the combined effects of all community members, with particular weight given to abundant taxa. However, significant differences in relative abundance may exist in comparisons of individual taxa between treatments that are not discernible when effects of whole communities are aggregated. To test for differences in the relative abundances of individual taxa between treatment levels (contrasts of host ploidy and host genotype), we used a hierarchical Bayesian modeling approach reliant upon the Dirichlet and multinomial distributions (60, 61). The Dirichlet-multinomial model (DMM) accounts for the compositional nature of community sampling (whether in sequencing data or any finite number of taxa observations from an assemblage), allows information sharing among replicates within a category, and obtains estimates of the relative abundance of each microbial taxon, while propagating uncertainty in those estimates. We estimated differences in the relative abundance of each microbial taxon between experimental treatments through comparison of parameter estimates as per Harrison et al. (60).

Briefly, DMM estimates the multinomial parameters describing the relative abundances of each taxon in a replicate, designated a vector of parameters (*p*). These multinomial parameters were informed by a Dirichlet distribution, with parameters characterizing the expected frequencies of each taxon (π), where π is a vector describing the expected relative abundance of each taxon in the sampling group (60, 62). To quantify differences in the relative abundance of taxa between groups, we calculated the posterior probability distribution for the difference in π_i_ parameters between groups for taxon *i*. The probability of an effect of treatment on a focal taxon can be determined by the location of zero in this distribution of differences. Following convention, if 95% or more of the distribution did not overlap zero, then there was little evidence that the relative abundance of the focal taxon differed between treatment groups.

The DMM offers several advantages over existing analytical methods. First, parameters were estimated while propagating uncertainty, thus avoiding cumbersome multiple-comparison correction and precluding the use of *P* values. Second, information was shared among replicates and treatment groups. Third, rarefaction is unnecessary because analyses are performed on proportions, which are estimated using information from all replicates within a sampling group. Fourth, the Dirichlet and multinomial distributions have interdependent parameters that reflect the compositional nature of sequencing data; thus, we modeled the composition as a whole as opposed to estimating the relative abundance of each taxon separately.

DMM was specified in the Stan probabilistic programming language and implemented through the rstan (ver. 2.18.2) package in the R statistical computing environment (ver. 3.6.0) (63, 64). For each of four chains, the sampler was run for 1,500 steps as a burn-in period and was followed by an additional 1,000 steps (a total of 4,000 samples were drawn from the posterior distributions of focal parameters [π from each sampling group]). The Gelman-Rubin statistic was computed to measure convergence among chains (65). Separate models were constructed for the following comparisons: all diploid versus all tetraploid hosts, Columbia versus Landsberg erecta, and Columbia tetraploid versus all other treatments (pooled).

For taxa that differed credibly among our treatment groups, we used NCBI BLAST to confirm 16S rRNA identifications generated using the SILVA database (66, 67). We report species-level identification in cases where reads align 100% with entries in the 16S rRNA sequence database and report the bacterial family if genus-level resolution was not corroborated by SILVA (68).

To assess effects on plant performance in experiment 2, we used fixed-effect, three-way ANOVA, where inoculum, genotype, and block were the explanatory variables. For purposes of data representation, residuals were plotted after statistically accounting for the effect of genotype and block. Finally, we used planned comparisons to contrast plant performance between plants grown in the Col-2x versus Col-4x inocula and Ler-2x versus Ler-4x inocula.

### Data availability.

Raw sequences were uploaded to the NIH NCBI Short Read Archive under project ID PRJNA474006. Code for all analyses is available via the Dryad database (DOI: 10.5061/dryad.hqbzkh1ff).
