# Supplementary material for: Whole-Genome Duplication and Host Genotype Affect Rhizosphere Microbial Communities
Source: mSystems. 2022 Jan 11;7(1):e00973-21. doi: 10.1128/msystems.00973-21 (PMC8751390; doi:10.1128/msystems.00973-21)
Supplement: TABLE S1 [file msystems.00973-21-st001.docx]

**Supplemental Table 1**

| **Processing Step** | **Number of Reads** |
| --- | --- |
| Initial | 3 282 410 |
| Filtering and Trimming, Sample Inference, Chimera Removal | 1 985 490 |
| Removal of SILVA identified Chloroplast, Mitochondrial, Eukaryotic DNA | 1 879 090 |
| Removal of Bacteria associated with unplanted soil matrix samples | 1 647 741 |
