# Supplementary material for: Whole-Genome Duplication and Host Genotype Affect Rhizosphere Microbial Communities
Source: mSystems. 2022 Jan 11;7(1):e00973-21. doi: 10.1128/msystems.00973-21 (PMC8751390; doi:10.1128/msystems.00973-21)
Supplement: TABLE S2 [file msystems.00973-21-st002.docx]

**Supplemental Table 2**

| **Trait** | **P - Value** | **Mean** | **SE** |
| --- | --- | --- | --- |
| Aboveground biomass | < 0.001 | 13.97 g | 0.74 |
| Belowground biomass | < 0.001 | 45.38 g | 3.52 |
| Root : Shoot | **ns** | 4.32 | 0.30 |
| Days to bolting | **ns** | 26 days | 0.24 |
| Days to flowering | **ns** | 41 days | 2.36 |
| Rosette Diameter | **ns** | 14.04 mm | 0.27 |
